# Reply to “Comment on Ahmad, I.M. et al. Healthcare Workers Occupationally Exposed to Ionizing Radiation Exhibit Altered Levels of Inflammatory Cytokines and Redox Parameters. *Antioxidants*, 2019, *8*, 12”

**DOI:** 10.3390/antiox8020043

**Published:** 2019-02-16

**Authors:** Iman M. Ahmad

**Affiliations:** Department of Medical Imaging and Therapeutic Sciences, College of Allied Health Professions, University of Nebraska Medical Center (UNMC), Omaha, NE 68198, USA; iman.ahmad@unmc.edu; Tel.: +1-402-559-6911

Bevelacqua and Mortazavi [1] questioned the usefulness of our published paper, which addresses an important topic regarding the effect of radiation on cytokines and redox parameters in occupationally-exposed radiation healthcare workers. Although some of the concerns of Bevelacqua and Mortazavi are valid, particularly their point regarding the limited sample sizes in our study, we believe that it is critical for us to address some of the other so-called “shortcomings”. A point-by-point response to the commentary authored by Bevelacqua and Mortazavi is provided below.

Low-intensity radiofrequency radiation (RFR) is classified as non-ionizing radiation and we agree that for most people, RFR likely accounts for the majority of their radiation exposure. However, our study focused on occupationally-exposed radiation healthcare workers, specifically radiologic technologists. It should be noted that these healthcare workers are exposed to ionizing radiation in their work environment. Although the review paper by Yakymenko et al. indicates that RFR induces “oxidative effects” in biological systems, we speculate that the effect of ionizing radiation on redox parameters is greater than that from non-ionizing radiation. In addition, it must be noted that the control subjects in our study work in the same hospital and live in the same region as the radiologic technologists. Thus, it is likely that both groups are exposed to similar levels of the low-intensity and non-ionizing RFR. 

Alcohol consumption for our study subjects ranged from 1–12 days/month of having at least one standard drink. There was no significant interaction between alcohol consumption and study groups as shown in Table 1. With regard to the “great heterogeneity of the samples”, all radiation-exposed healthcare workers in our study are radiologic technologists who perform medical imaging procedures, including conventional radiography, interventional radiography or computed tomography, as mentioned in the methodology of our paper. Importantly, all of these imaging procedures use ionizing radiation in the form of x-rays and thus, there was no mixed population. Furthermore, none of our participants work in radiation therapy. With regards to the sample size, as shown in all the figures, we are reporting the difference between all exposed and unexposed subjects. Although the sample size is small for each subgroup, we feel that the subgroup data provide at least some insight into the cause of the differences in unexposed versus exposed individuals. 

As mentioned above, our study participants were exclusively radiologic technologists who perform medical imaging procedures, including conventional radiography, interventional radiography or computed tomography. All use ionizing radiation (x-rays). None of our participants work in radiation therapy where they would use procedures with different types of ionizing radiation, such as gamma-rays, protons, heavy ions or neutrons. Furthermore, and as mentioned above, there was no “mixing of worker types”, that is, our study participants did not include radiologists, medical physicists, nurses or other support personnel. Thus, this comment by Bevelacqua and Mortazavi is not relevant to our published study.

In summary, the primary goal of our study was to improve our understanding of the long-term health effects of ionizing radiation, specifically x-rays, on radiologic technologists performing medical imaging procedures, including conventional radiography, interventional radiography or computed tomography. We believe that the conclusions we made in our published study are supported by the data presented therein. 
